# Supplementary material for: Probing the Drug Dynamics of Chemotherapeutics Using Metasurface-Enhanced Infrared Reflection Spectroscopy of Live Cells
Source: Cells. 2022 May 10;11(10):1600. doi: 10.3390/cells11101600 (PMC9139550; doi:10.3390/cells11101600)
Supplement: Supplementary file 1 [file cells-11-01600-s001.zip › cells-1697054-supplementary.pdf]

Supplementary Materials

Probing the Drug Dynamics of Chemotherapeutics Using Metasurface-Enhanced Infrared Reflection Spectroscopy of Live Cells

First, we examined the unshifted protein time-dependent signals in Figure S1 (a). There were five replicates of the TRIP-treated cell experiments. Each has a differently timed two protein spikes. We didn't see an obvious pattern of the amplitude of the two spikes. TRIP 5 has the earliest first protein spike at t = 89 min, while the C≡O stretching half-max arrived at t= 85 min. Hence, we note its latency of the first protein spike as 4 minutes. We noted the latency of every replicate in Figure S1 (b). We found a consistent latency of 4-6 minutes for every replicate. Next, we shifted the time series of TRIP 1-4 to match their C≡O stretching half-max signals to that of TRIP 5. We performed the same tasks for every spectral window (protein, lipid, plasmonic and C≡O stretching). The calibrated protein time-dependent signals are shown in Figure S1 (c). The difference between the unshifted and calibrated signals is very clear visually. The calibrated signals have a smaller spread in trajectory and therefore smaller standard errors.

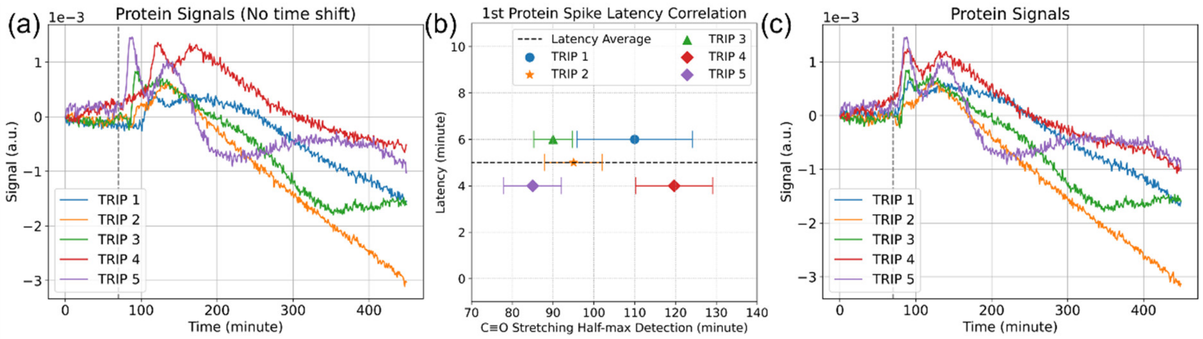

Figure S1. The timing calibration of biological signals in TRIP-treated group by the C≡O stretching signals. **(a)** The unshifted protein signals. The gray dashed line marked the mean time to detect the start of increase in the C≡O stretching signals at t_0 = 70 min. (Defined as the drug arrival time for convenience.) **(b)** The correlation of C≡O stretching signals and first protein spikes. The C≡O stretching signals arrived a few minutes earlier than the protein signals. Each replicate's time series is calibrated to the C≡O stretching half-max at t = 85 min (TRIP 5). The figure is identical to Figure 3 **(c)**, but each replicate is marked specifically. **(c)** The calibrated protein signals. The gray dashed line marked the drug arrival time.

Proper statistics can be derived from the aligned time series. In Figure S2, we show the calibrated TRIP-treated cells' protein and plasmonic resonance signals individually against the control's protein signals (shown as an average curve with shaded standard error for simplicity). We can see that the standard errors were small and consistent in the control. This was to check the reliability of MEIRS. The calibrated TRIP-treated has larger spread in standard errors than that of the control. This justifies the use of Welch's test (unequal variance t-test). We performed Welch's test for the mean signal of each group in each spectral window at each minute and obtained the p-value at each minute. In Figure 5(c), we plotted the p-values as a time series for the protein window. The statistical significance can be established as soon as t = 220 min.

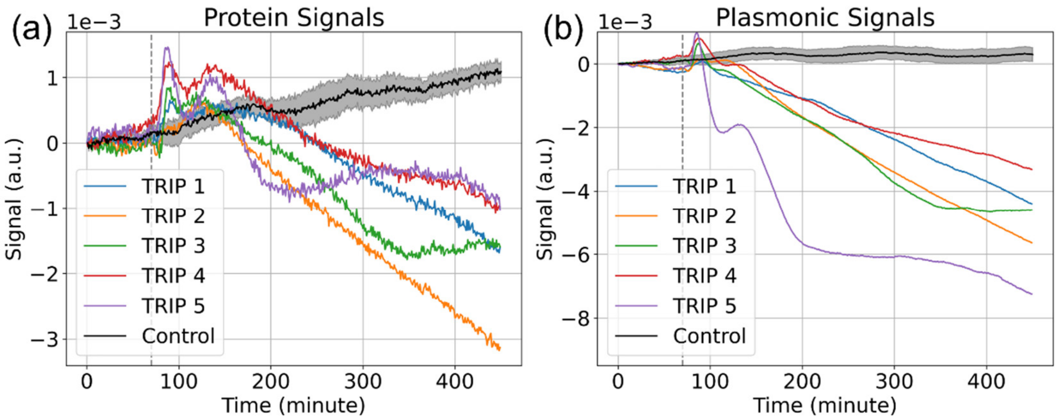

Figure S2. The calibrated time series of TRIP-treated group (5 replicates, shown individually) compared to the control group (5 replicates, shown as an average curve with shaded standard errors) in the **(a)** protein and **(b)** plasmonic spectral window.
